# Stability of tuberous sclerosis complex 2 is controlled by methylation at R1457 and R1459

**DOI:** 10.1038/s41598-020-78274-6

**Published:** 2020-12-03

**Authors:** Seishu Gen, Yu Matsumoto, Ken-Ichi Kobayashi, Tsukasa Suzuki, Jun Inoue, Yuji Yamamoto

**Affiliations:** Laboratory of Nutritional Biochemistry, Department of Agricultural Chemistry, Faculty of Applied Bioscience, Tokyo University of Agriculture, 1-1-1 Sakuragaoka, Setagaya-ku, Tokyo, 156-8502 Japan

**Keywords:** Biochemistry, Cancer

## Abstract

Mutations in genes that encode components of tuberous sclerosis complex 2 (TSC2) are associated with tuberous sclerosis complex disease. TSC2 interacts with tuberous sclerosis complex 1 to form a complex that negatively regulates cell growth and proliferation via the inactivation of mechanistic target of rapamycin complex 1. The activity of TSC2 is mainly regulated via posttranslational modifications such as phosphorylation. However, the control of TSC2 activity is not entirely achieved by phosphorylation. In this study, we show that TSC2 is methylated at R1457 and R1459 by protein arginine methyltransferase 1 (PRMT1). Methylation of these two residues can affect the phosphorylation status through protein kinase B (Akt) of TSC2 at T1462 and is essential for TSC2 stability. Taken together, these findings indicate that novel posttranslational modifications are important for the regulation of TSC2 stability through PRMT1-mediated methylation.

## Introduction

Tuberous sclerosis complex (TSC) is an autosomal dominant disorder that is characterized by benign tumours in the brain and other vital organs and is due to mutations in the tumour suppressor gene tuberous sclerosis complex 2 (*TSC2*). In addition to *TSC2* mutations, patients with TSC exhibit loss of TSC2 protein expression and concurrent increases in cell growth and proliferation^1,2^. TSC2 is expressed in lysosomes, where it interacts with tuberous sclerosis complex 1 (TSC1) to form a complex that has Rheb GTPase-activating protein (GAP) activity that negatively regulates cell growth through the inactivation of its mechanistic target, mechanistic target of rapamycin complex 1 (mTORC1)^3^. Our group and other groups have recently shown that TSC2 is a multifunctional protein that can also control other pathways, such as those related to Rab5^4^ and Rac1 ^5,6^.

TSC2 activity is regulated by insulin signalling ^7^, energy stress ^8,9^, oxygen pathways, and growth factors ^3^, which all inactivate TSC2 by promoting posttranslational modifications such as phosphorylation. The serine/threonine kinase protein kinase B (Akt) is a master regulator of survival, apoptosis, and cell growth ^10^ that is activated by insulin and various growth factors. Akt phosphorylates TSC2 at a common recognition motif, RxRxxS/T (R, arginine; S, serine; T, threonine; x, any amino acid), that is located at multiple sites, including S939 and T1462, of human TSC2 ^7,11,12^.

In addition to phosphorylation, in general, posttranslational modifications (such as palmitoylation, acetylation, ubiquitination, and methylation) can regulate protein activity ^3^. For methylation in particular, protein arginine methyltransferases (PRMTs) catalyse the addition of methyl residues from *S*-adenosyl methionine (SAM) to guanidino nitrogen atoms of arginine residues ^13^. In mammalian cells, 10 PRMTs are responsible for arginine methylation and have been classified into three groups, namely, monomethylarginine (MMA: type 1), asymmetric dimethylarginine (ADMA: type 2), or symmetric dimethylarginine (SDMA: type 3) ^14^. Protein arginine methyltransferase 1 (PRMT1) belongs to the type 1 enzyme group, which is ubiquitously expressed and preferentially recognizes glycine-arginine rich (GAR) motifs (RGG/RG motif; R, arginine; G, glycine) on a target protein. PRMT1 regulates a wide variety of cellular processes, such as transcription, DNA repair, and transcriptional regulation ^14–16^ and is also a major methyltransferase ^11^ that is linked to diseases such as cancer and metabolic disorders ^14^.

In this study, we report that PRMT1 methylates TSC2 at R1457 and R1459. The methylation status of the TSC2 protein inhibits phosphorylation at T1462, leading to the regulation of its stability.

## Results

### PRMT1 methylates TSC2 at R1457 and R1459 in vitro

**Figure 1:**
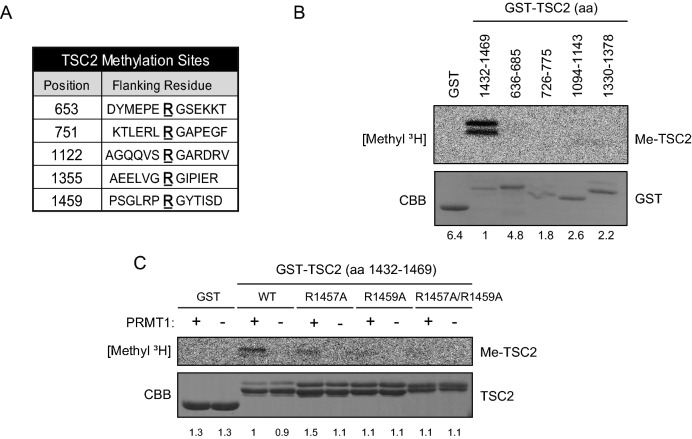
In vitro methylation assay of PRMT1-mediated methylation of TSC2 at R1457 and R1459. (**A**) Predicted arginine methylation sites in TSC2 determined using the iPTMnet search tool. (**B**) Methylation at predicted sites analysed by incubating GST-TSC2 fusion peptides with [^3^H]-SAM and recombinant PRMT1. Methylation was detected by autoradiography. The CBB staining for each GST-TSC2 peptide was quantified by ImageJ. (**C**) Confirmation of TSC2 methylation at R1457 and R1459 of TSC2 by analysis of methylation of the wild-type GST-TSC2 (aa 1432–1469) and mutated peptides having single (R1457A or R1459A) or double (R1457A/R1459A) arginine to alanine substitutions performed as described for (**C**). CBB: Coomassie Brilliant Blue. The CBB staining for each GST-TSC2 peptide was quantified by ImageJ.

To investigate whether TSC2 could be methylated, we first searched for an RGG/RG methylation target motif using the posttranslational modification site search tool iPTMnet (https://research.bioinformatics.udel.edu/iptmnet/). This search identified R653, R751, R1122, R1355, and R1459 in TSC2 as candidate methylation sites (Fig. 1A). To evaluate the methylation possibility of these candidate sites, we then expressed and purified corresponding fusion peptides with glutathione S-transferase (GST): GST-TSC2 (aa 636–685), GST-TSC2 (aa 726–775), GST-TSC2 (aa 918–967), GST-TSC2 (aa 1094–1143), GST-TSC2 (aa 1330–1378), and GST-TSC2 (aa 1432–1469) from bacteria (Fig. 1B). These fusion peptides, which contained candidate methylation sites, were used in the following methylation assays. Initially, we tested PRMT1 because it is a predominant protein methylation enzyme in mammalian cells. We incubated the TSC2 peptides with *S*-adenosyl-l-methyl-[^3^H] methionine ([^3^H]-SAM) as a methyl donor in the presence of recombinant PRMT1. Obvious methylation was observed for the peptide containing R1459 [GST-TSC2 (aa 1432–1469)] (Fig. 1B). Furthermore, the methylation assays indicated that among the known type 1 PRMTs, only PRMT1 could catalyse methylation of the GST-TSC2 (aa 1432–1469) fusion peptide (Supplementary Fig. 1).

Although a typical PRMT1 methylation RGG/RG motif was not present in this GST-TSC2 (aa 1432–1469) peptide, an RXR motif, which has been proposed as another PRMT1 target sequence, was present in the peptide ^17^. To confirm the methylation site in the GST-TSC2 (aa 1432–1469) peptide, we replaced both R1457 and R1459 with alanine. In the following methylation assays using recombinant PRMT1, the methylation levels of these mutated peptides were decreased compared to that observed for the wild-type GST-TSC2 (aa 1432–1469) peptide (Fig. 1C). These methylation sites are highly conserved among several species (Supplementary Fig. 2). Therefore, our data indicate that R1457 and R1459 are primary targets for PRMT1 methylation, although other arginine residues could also be methylated by other PRMTs. These results show the existence of other regulatory mechanisms of TSC2 activity that involve the methylation of TSC2 arginine residues.

**Figure 2:**
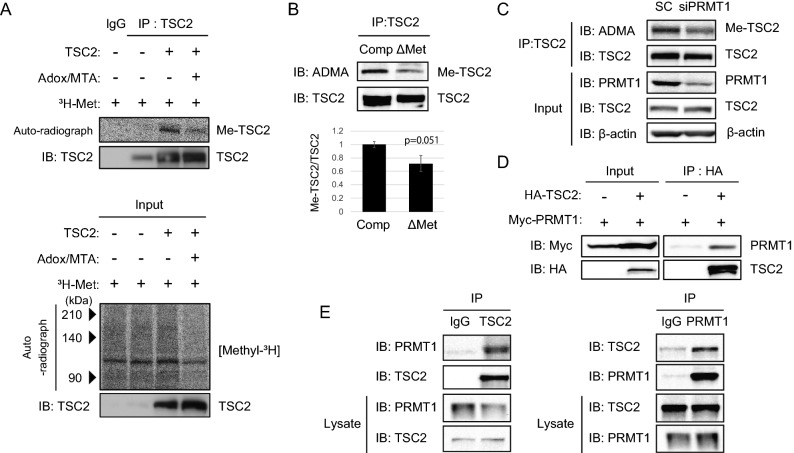
*PRMT1* binds and methylates intact TSC2. (**A**) Autoradiography and western blot analysis. Methylation of intact TSC2 in 293 T cells expressing wild-type TSC2-overexpressing CHX (40 μg/μL) and chloramphenicol (40 μg/μL) cultured in medium containing [^3^H]-Met with or without the PRMT1 inhibitor ADOX/MTA (20 µM). TSC2 was immunoprecipitated (IP) and TSC2 methylation was detected by autoradiography. Levels of precipitated TSC2 were assessed by western blotting (IB; left panel). The effect of MTA was confirmed by measuring total protein methylation (upper panel), and TSC2 expression was confirmed by IB (right panel). (**B**) Lysates from 293 T cells treated with complete media (comp) or methionine deprivation media (ΔMet) were immunoprecipitated and immunoblotted with an anti-TSC2 antibody or anti-dimethyl arginine antibody (ADMA). (**C**) Lysates from 293 T cells transfected with Myc-PRMT1 and HA-TSC2 were immunoprecipitated with anti-HA antibody and subsequently immunoblotted with anti-Myc antibody. (**D**) Binding of TSC2 to PRMT1 was confirmed using 293 T cells transfected with Myc-PRMT1 and HA-TSC2. Cell lysates were immunoprecipitated with an anti-HA antibody and subsequently immunoblotted with an anti-Myc antibody. (**E**) Lysates from 293 T cells were immunoprecipitated with the anti-PRMT1 antibody or anti-TSC2 antibody and subsequently immunoblotted with anti-TSC2 antibody or anti-dimethyl arginine antibody.

### PRMT1 binds and methylates intact TSC2 in cells

We next analysed whether the full-length TSC2 protein is methylated by PRMT1 in cells. Initially, to determine the methylation status of TSC2, TSC2-overexpressing 293 T cells were incubated with [^3^H]-methionine in the presence or absence of the PRMT inhibitors ^18^ adenosine-2′,3′-dialdehyde (Adox) and 5′-deoxy-5′-(methylthio)adenosine (MTA) before the cells were harvested, and TSC2 was immunoprecipitated. The decrease in total protein methylation was first confirmed by Adox and MTA treatments (Fig. 2A, lower panel). Under this condition, arginine methylation in TSC2 decreased in the presence of these PRMT inhibitors (Fig. 2A, upper panel). We further determined and analysed the TSC2 methylation status of cells incubated in the absence of methionine by western blotting using a specific ADMA antibody. As shown in Fig. 2B, methionine depletion reduced the methylation status of TSC2, suggesting that methylation occurs in intact TSC2 under physiological conditions. We then examined the methylation status of TSC2 under the condition of reduced expression of PRMT1 via siRNA in 293 T cells. In cells transfected with siPRMT1, we observed decreased amounts of TSC2 arginine methylation, suggesting a PRMT1-mediated TSC2 methylation mechanism (Fig. 2C).

We then examined whether TSC2 methylation occurs via the interaction of PRMT1 with TSC2. Hemagglutinin (HA)-TSC2 co-expressing Myc-PRMT1 cells were prepared, and TSC2 was immunoprecipitated using an anti-HA antibody. As shown in Fig. 2D, TSC2 and PRMT1 precipitated as a complex, suggesting that the PRMT1 interaction is important for TSC2 methylation. We also examined whether intact TSC2 also interacted with PRMT1. TSC2 coimmunoprecipitated with PRMT1 (Fig. 2E, left panel) and vice versa (Fig. 2E, right panel).

Thus, we identified PRMT1 as a novel binding partner of TSC2, and together with the data shown in Fig. 1, we hypothesized that PRMT1 methylates full-length TSC2 at R1457 and R1459.

### Methylation of TSC2 at R1457 and R1459 blocks Akt-dependent TSC2 phosphorylation at T1462

R1457 and R1459 on TSC2 partially overlap with the phosphorylation site of TSC2 via the Akt phosphorylation motif (RxRxxS/T), which is highly conserved in many species, including *Homo sapiens*, *Mus musculus*, and *Rattus norvegicus* (Supplementary Fig. 2). This phosphorylation modification via Akt controls TSC2 activity ^7,12^.

**Figure 3:**
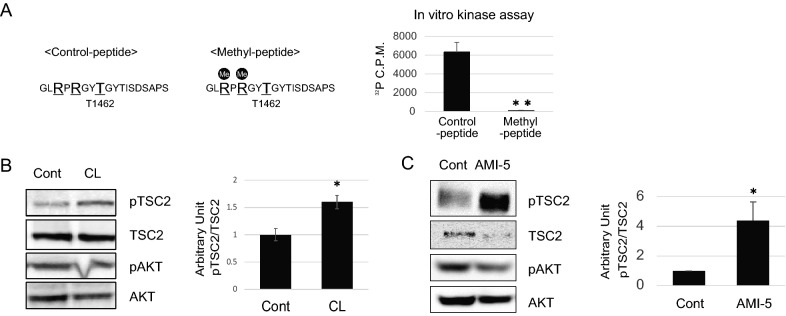
Arginine methylation of TSC2 blockes Akt-dependent TSC2 phosphorylation at T1462. (**A**) Phosphorylation assays were conducted using the unmodified TSC2 peptide (cont) and the modified arginine methylated peptide (Methyl). Peptides were incubated with recombinant Akt and [γ-^32^P] ATP and phosphorylation was detected using a scintillation counter. Error bars indicate standard deviation (SD) from three independent experiments; **p < 0.01. (**B/C**) Effect of TSC2 methylation on TSC2 phosphorylation measured by inhibiting (**B**) [^3^H]-SAM using cycloleucine (100 mM) with MG132 (10 µM) or (**C**) PRMT1 activity using AMI-5 (2.0 µM). Phosphorylated TSC2 (T1462) and AKT (S473) levels were analysed by western blotting. Error bars indicate the SD from two independent experiments; *p < 0.05. All experiments were repeated three times.

Therefore, we first investigated whether methylation at R1457 and R1459 could affect Akt-mediated phosphorylation using in vitro kinase assays. Two TSC2 peptides spanning residues aa 1455–1469, an unmodified TSC2 peptide (control peptide), and a TSC2 peptide that was methylated at R1457 and R1459 (methyl peptide) were synthesized and subjected to an in vitro kinase assay. Recombinant Akt phosphorylated the control TSC2 peptide at T1462, whereas phosphorylation of the methyl peptide was remarkably reduced (Fig. 3A). Moreover, in 293 T cells, Akt-mediated phosphorylation of TSC2 at T1462 was increased after treatment with cycloleucine (CL; an inhibitor of the SAM synthase MAT2A)^19^ and eosin Y disodium trihydrate (AMI-5, an inhibitor of PRMT1) ^20^ (Fig. 3B,C). These results indicate that TSC2 phosphorylation at T1462 is partially controlled by the methylation status of TSC2.

Previous reports also revealed that insulin stimulation promotes TSC2 phosphorylation by Akt, which leads to acute dissociation of TSC2 from the lysosomal surface ^21,22^. To determine whether the inhibition of PRMT1 prevents the lysosomal localization of TSC2, we performed immunofluorescence analyses using an anti-TSC2 antibody and a marker antibody against the lysosomal protein lysosomal-associated membrane protein 2 (LAMP2). Extensive TSC2–LAMP2 colocalization was significantly reduced by treatment with insulin or the PRMT1 inhibitor AMI-5 (Supplementary Fig. 4). Therefore, colocalization was affected by the hypomethylation of TSC2.

**Figure 4:**
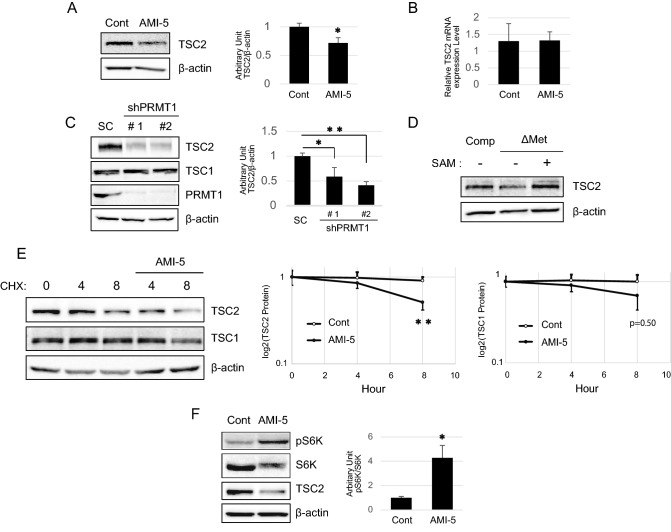
Increased stability of methylated TSC2. (**A**) Levels of TSC2 in 293 T cells with or without AMI-5 (2.0 µM) as assessed by western blotting. The error bars indicate the SD from two independent experiments; *p < 0.05. (**B**) Effect of AMI-5 (2.0 µM) on TSC2 mRNA expression levels in RT-qPCR analyses. Data represent expression levels of TSC2 mRNA normalized to β-actin mRNA levels. (**C**) TSC2 protein expression levels were determined in 293 T cells with PRMT1 expression knockdown using western blotting. Error bars indicate the SD from two independent experiments; **p < 0.01, *p < 0.05. Experiments were repeated three times. (**D**) Levels of TSC2 in 293 T cells with or without deprivation of methionine in the addition of SAM (100 µM) for 5 h as assessed by western blotting. (**E**) Half-life of TSC2 analysed by treating 293 T cells with AMI-5 (2.0 µM), followed by the protein synthesis inhibitor CHX (10 µg/mL). Cell lysates were directly subjected to western blotting 4 or 8 h after the treatments. Error bars indicate the SD from two independent experiments; **p < 0.01. All experiments were repeated three times. (**F**) Effect of inhibiting methylation on the activity of TSC2 measured after treatment of cells with AMI-5 (2.0 µm) and CHX (10 µg/mL) for 5 h. The activity of target of mTORC1 was assessed using phospho-S6K (T389) levels. Error bars indicate SD from three independent experiments; *p < 0.05.

### Arginine methylation of TSC2 is important for its stabilization

As shown in Fig. 2B, when cells were treated with AMI-5, we observed a decrease in TSC2 protein levels compared to the control, and we hypothesized that TSC2 stability may be affected through PRMT1 methylation. To determine if TSC2 methylation protects against protein degradation, we first analysed the expression levels of the TSC2 protein in the presence of AMI-5. Treatment with AMI-5 decreased the TSC2 protein levels (Fig. 4A), whereas the levels of TSC2 mRNA were not affected (Fig. 4B). To confirm that PRMT1 was involved in this mechanism, PRMT1 expression was reduced in cells through shPRMT1, and a significant reduction in TSC2 protein expression was observed, as shown in Fig. 4C. Furthermore, as shown in Fig. 2B, removing methionine from the medium led to hypomethylation of TSC2; therefore, as expected, under this condition, decreased TSC2 expression was observed, and adding the methyl donor SAM rescued the expression of TSC2 (Fig. 4D). To confirm that the protein stability was affected during hypomethylation of TSC2, cells were treated with AMI-5 in combination with the protein synthesis inhibitor cycloheximide (CHX). As shown in Fig. 4E, these treatments led to a significant downregulation of TSC2 after 8 h. However, TSC1 was not significantly affected.

Finally, we investigated whether decreases in TSC2 activity could affect downstream mTORC1 activity, which was measured based on phospho-S6K levels (T389). PRMT1 inhibitor-induced TSC2 degradation promoted S6K phosphorylation (Fig. 4F). Collectively, these results showed for the first time that TSC2 is stabilized by methylation. Furthermore, mTORC1 activity was partially regulated by PRMT1-mediated TSC2 methylation.

Therefore, these data indicate that PRMT1-mediated methylation is required for the stability of TSC2 and further affects downstream signal transduction, i.e., mTORC1 activity.

## Discussion

TSC2 is a tumour suppressor protein that is responsible for the autosomal dominant disorder TSC. The activity of TSC2 is mainly controlled through phosphorylation at various sites across the protein. In addition to phosphorylation, TSC2 activity is modulated by other posttranslational modifications, such as palmitoylation, acetylation and ubiquitination ^3^.

In this study, we showed that in addition to these posttranslational modifications, TSC2 is also methylated. By using wild-type and mutant TSC2 peptides, we identified the methylation sites as R1457 and R1459. In humans, there are ten known PRMTs, and PRMT1 belongs to the type 1 enzyme group and is the main enzyme responsible for the generation of dimethylarginines in proteins. Although the GAR motif (RGG/RG motif) is the preferred target for PRMT1, it can also target the RXR motif ^17,23^, which is present in TSC2 at R1457-R1459. Although we observed a weak band in the TSC2 R1457A mutant peptide (Fig. 1C), we need to further analyse the possibility of R1457 methylation at TSC2. Furthermore, intact TSC2 methylation was observed in the autography and western blotting analysis and inhibited by using Adox and MTA (Fig. 2A). Furthermore, siPRMT1 reduced the methylation status of TSC2, and this methylation occurred by TSC2 interacting with PRMT1. Thus, we report for the first time that PRMT1 is a novel TSC2 binding partner that methylates TSC2 at R1457 and/or R1459.

TSC2 activity is mainly regulated through phosphorylation via Akt and 5′ AMP-activated protein kinase (AMPK) at multiple sites ^3^. In particular, multiple sites of the Akt phosphorylation motif (RxRxxS/T) have been identified on TSC2; however, only two of these sites are conserved and phosphorylated in *Drosophila* at S939 and T1462. TSC2 lacking these two conserved sites can dominantly inhibit insulin-stimulated mTORC1 activity ^24^. The methylation sites at R1457 and R1459 overlapped with the Akt phosphorylation site, suggesting that TSC2 methylation could affect its phosphorylation status and in turn its activity. Indeed, methylation of FOXO1 at R248 and R250 by PRMT1 has been reported to interfere with phosphorylation at S253 and control its activity ^25^. Our finding that methylation at TSC2 R1457 and R1459 inhibited its phosphorylation led to the hypothesis that the methylation of TSC2 interferes with Akt-dependent phosphorylation at T1462.

Plas et al. have shown that Akt results in a decrease in the total protein levels of TSC2 and FOXO3a. In this report, they showed that the Akt-stimulated decrease in TSC2 correlated with a decrease in the protein level of its binding partner TSC1 ^26^. In our report, we also observed that hypomethylation decreased the protein level of TSC2, and further analysis revealed that TSC2 phosphorylation at the T1462 site was increased. Thus, we propose here that PRMT1 methylation blocked phosphorylation via Akt at T1462. In addition to T1462, Akt also phosphorylates TSC2 at S939 ^7,12^ and S981 ^27^. A study by Cai et al. showed that phosphorylation at these sites by Akt promotes the cytosolic localization of TSC2, where 14–3–3 is able to bind ^21^, but they did not examine phosphorylation at T1462. We did not observe methylation of R934 and R936 (data not shown), suggesting that the Akt motif at S939 could be protected by 14–3–3 to preclude methylation by PRMT1. The physiological implications of phosphorylation of T1462 are uncertain, but our findings suggest a novel PRMT1-mediated methylation modification mechanism.

Cells transfected with siRNA for PRMT1 or treated with the PRMT1 inhibitor AMI-5 had decreased the expression of the TSC2 protein (Fig. 4C,D). The finding that TSC2 mRNA levels were preserved in AMI-5-treated and methionine-depleted cells (data not shown) indicates that the reduction in TSC2 is not due to changes in TSC2 gene expression arising from a PRMT1-mediated chromatin modification caused by the methylation of R3 in histone H4 ^28^. Furthermore, the TSC2 protein level in cells treated with AMI-5 in the absence of CHX was significantly decreased compared with that in untreated cells (Fig. 4E). Thus, we hypothesized that the stability of TSC2 is associated with PRMT1-mediated methylation. To date, a specific TSC2 phosphatase has not been reported, and we suggest that controlling the phosphorylation of TSC2 at T1462 via methylation may be a novel regulatory mechanism to control TSC2 activity.

Our findings suggest that hypomethylation leads to TSC2 degradation. We have identified several methylation sites that are not candidates for PRMT1 but may be methylated through other PRMTs, including the putative arginine methyltransferase NADH:ubiquinone oxidoreductase complex assembly factor 7 (NDUFAF7) ^29^. Thus, mutations in these arginine residues may be important for the function of TSC2, presumably its stability. Loss of heterozygosity in TSC2 has been documented in tumours from TSC patients, and the complete loss of TSC2 protein is the main cause of TSC ^2^. However, we have previous reported that the carbohydrate metabolism is affected in the TSC2 heterozygote animal, Eker rat (TSC2±) ^30^. These findings led to the conclusion that partial loss of TSC2 protein may cause metabolic stress and influence to the cause of TSC and giving ketogenic diet to these Eker rat have also increase the tumor development ^31^.

Therefore, mutations in TSC2 may lead to unstable TSC2 and therefore led to the development of TSC tumours. Indeed, Guan et al*.* showed that TSC patients with mutations of arginine residues in TSC2 have low GAP activity ^32,33^, indicating the importance of arginine residues for both GAP functions ^34^ and TSC2 activity. Notably, the National Institutes of Health (NIH) mutation database (URL: https://portal.gdc.cancer.gov/) includes TSC patients who carry mutations at arginine residues, including in R1457 and R1459 of TSC2, suggesting that these sites do have physiological relevance for TSC pathology.

In summary, we identified a novel posttranslational modification of TSC2 in the form of PRMT1-mediated methylation. This methylation promoted the stability of TSC2 by interfering with Akt-mediated phosphorylation at T1462. Inhibition of PRMT1 is often considered the target for treating cancer cells ^35^. In general, methionine restriction (MR), which reduces the methyl donor for PRMTs, has been well studied as inhibiting tumour development in various cancers. However, our data may suggest that PRMT inhibitors or MR may activate mTORC1, leading to tumour development in some patients. Therefore, treating TSC patients with PRMT1 inhibitors may have side effects, and specific activators may be useful alternative therapeutic treatments. However, the upstream signalling pathway that activates PRMT1 is still not clear. In addition, H_2_O_2_ and Ca^2+^ signals have been reported to enhance the activity of PRMT1 ^25,36^. Additional studies are needed to define the mechanism by which methylation at these residues could affect TSC disease pathology and whether activation or inhibition of PRMT1 could be an effective strategy to mitigate the symptoms of TSC disease.

## Materials and methods

### Antibodies and materials

The following primary antibodies were used and diluted in 5% BSA/TBST: anti-PRMT1 polyclonal antibody (Cell Signaling Technology (CST), Cat. #2449), anti-TSC2 monoclonal antibody (CST, Cat. #4308), anti-phospho-TSC2 (T1462) monoclonal antibody (CST, Cat. #3611), anti-β-actin monoclonal antibody (Santa Cruz, Cat. #sc-47778), anti-Akt polyclonal antibody (CST, Cat. #9272), anti-phospho-Akt (S473) monoclonal antibody (CST, Cat. #4051), anti-asymmetric di-methyl arginine motif (adme-R) MultiMab Rabbit mAb mix antibody (CST, Cat. #13522), anti-HA HA-7 monoclonal antibody (Sigma, Cat. #H2663), anti-FLAG M2 monoclonal antibody (Sigma, Cat. #F3165), and anti-Myc My3 monoclonal antibody (MBL International, Cat. #M192-3). Anti-rabbit IgG (Cat. #A0545) and anti-mouse IgG (Cat. #I5381) were purchased from Sigma. Dulbecco’s modified Eagle’s medium (DMEM, Cat. #041-29775) was purchased from Wako. Foetal bovine serum (FBS) was purchased from Gibco (Cat. #10270-106).

Methionine deprivation media or cysteine deprivation media was purchased from the Research Institute for the Functional Peptides Co., Ltd.

The PRMT1 inhibitor AMI-5 was purchased from Santa Cruz (Cat. #133410U). The nonselective PRMT inhibitors Adox and MTA were purchased from Sigma-Aldrich (Cat. #A7154 and Cat. #D5011, respectively). CHX (Cat. #C7698), chloramphenicol (Cat. #C0378), and CL (Cat. #A48105) were all purchased from Sigma-Aldrich. The proteasome inhibitor MG132 was obtained from Funakoshi (Cat. #133407-82-6).

### Plasmids

Human *PRMT1*, *PRMT2*, *PRMT3*, and *PRMT6* were inserted into pRK7-N-Myc vectors. Human *TSC2* was amplified from HeLa cDNA by PCR. The primer sets used in this experiment are listed in Table EV1.

### Cell culture and transfection

Human embryonic kidney (HEK) 293 T cells and HeLa cells were maintained in DMEM supplemented with 10% FBS and 1% penicillin–streptomycin. 293 T cells were transfected with tagged Myc-PRMT1, Myc-PRMT2, Myc-PRMT3 or Myc-PRMT6 with HA-TSC2 using Lipofectamine LTX Reagent (Thermo Fisher, Cat. #15338100). Samples were analysed at 48 h post-transfection.

### Viral infection

To knock down *PRMT1* expression, cells were transfected with a specific shRNA as reported previously, with minor modifications ^22^. For lentiviral infection, pLKO.1 shRNA vectors expressing shRNAs were transfected into HEK293T cells with the lentiviral packaging plasmids psPAX2 and pMD2. Viruses were collected 48 h after transfection and concentrated via ultracentrifugation at 4 °C for 1.5 h at 23,000 rpm. Cells were infected, selected for puromycin resistance, and analysed 7 days after infection. The primer sets used in this experiment are listed in Table EV2.

### siRNA

To knock down *PRMT1* expression, HEK293T cells were treated with siRNA as described previously using a scramble control (SC) siRNA, PRMT1 #1 siRNA (siPRMT1#1), or PRMT1 #2 siRNA (siPRMT1#2), which were purchased from Thermo Fisher Scientific (SC; AM4611, siPRMT1#1; AM16708-10000, and siPRMT1#2; AM16708-10091). Each siRNA was transfected using RNAiMAX Lipofectamine (Life Technologies/Invitrogen, 13778-150) according to the manufacturer’s instructions. Cells were treated with 50 nM siRNA for 48 h.

### Recombinant GST-TSC2 peptide purification

DH5α competent *Escherichia coli* cells were transfected with GST-labelled full-length proteins and mutant plasmids, grown to OD = 0.6, and induced with 0.5 mM isopropyl β-d-1-thiogalactopyranoside (IPTG) at 30 °C for 6 h. After centrifugation, cell pellets were lysed with sonication in buffer containing 50 mM Tris (pH 8.0), 5 mM ethylenediaminetetraacetic acid (EDTA), 10% glycerol, 0.5% NP-40, 50 mM NaCl, 1 mM phenylmethylsulfonyl fluoride (PMSF), and 1 mM dithiothreitol (DTT). Cell lysates were pulled down with glutathione Sepharose (GST-tagged proteins) at 4 °C overnight. Subsequently, beads were washed three times with wash buffer containing 50 mM Tris-HCl (pH 7.4), 5 mM EDTA, 150 mM NaCl, and 0.5% Triton X-100, and bound proteins were eluted using elution buffer containing 50 mM Tris (pH 8.0) and 6.5 mM glutathione. The eluted proteins were then dialysed against a buffer containing 10 mM Tris (pH 7.4), 50 mM NaCl, 0.5 mM EDTA, and 0.05% 2-mercaptoethanol. The primer sets used in this experiment are listed in Table EV3.

### Autoradiography and western blot analysis

Autoradiography and western blot analysis of TSC2 were performed as described previously, with minor changes ^18^. Briefly, mock or TSC2-overexpressing HEK293T cells with cycloheximide (40 µg/µL) and chloramphenicol (40 µg/µL) cultured in medium containing [^3^H]-SAM with or without the PRMT1 inhibitor Adox/MTA (40 µg/µL) were pre-treated with methionine-free medium and incubated in the presence of l-[methyl-^3^H] methionine with/without the PRMT inhibitors Adox (40 µg/µL) and MTA (40 µg/µL). Immunoprecipitated TSC2 was detected by western blot analysis and autoradiography using a BAS scanner (BAS-2500, Fujifilm Co., Ltd.).

### In vitro methylation assay

In vitro methylation of TSC2 was assessed as reported previously, with minor changes ^25^. Briefly, 15 µg of purified recombinant GST-TSC2 (aa 1432–1470) was incubated with 1 ng GST-PRMT1 (BPS, Cat. #51041) or immunoprecipitated with Myc-PRMT1 in the presence or absence of [^3^H]SAM (*S*-[methyl-^3^H]; 5–15 Ci/µmol, ARC Inc.) in a total volume of 40 µl with phosphate-buffered saline (PBS) containing 50 mM Tris-HCl (pH 7.4), 5 mM EDTA, 150 mM NaCl, and 0.5% Triton X-100 at 30 °C for 2 h. Reaction products were resolved using Coomassie brilliant blue (CBB) staining and detected by autoradiography using a BAS-2500 scanner.

### In vitro kinase assay

In vitro kinase assays with Akt were performed as reported previously, with minor changes ^25,37^. Briefly, 500 µM TSC2 peptides were incubated in 500 µM MgCl_2_ before 100 ng of recombinant Akt was added (Thermo Fisher) together with 1 µCi of [γ-^32^P] ATP solution (Perkin Elmer) and 25 mM ATP (Sigma) to a final volume of 25 μl in H_2_O. Reaction mixtures were incubated in microcentrifuge tubes at 30 °C for 20 min. Subsequently, 15 μl aliquots of the mixture were loaded onto phosphor-cellulose paper discs (GE Healthcare, Cat. #10402506), which were washed three times for 20 min each in 1% phosphoric acid (pH 7.0; Wako, 168–27155). Relative activities were measured using a scintillation counter (LSC-5100, Aloka, Inc.). The methyl-TSC2 peptide (methyl peptide) GL(mR)P(mR)GYTISDSAPS and the unmodified TSC2 peptide (control peptide) GLRPRGYTISDSAPS were synthesized by SCRUM, Inc.

### Western blotting and immunoprecipitation analyses

Whole-cell lysates were resolved by 6–12% sodium dodecyl sulfate–polyacrylamide gel electrophoresis (SDS-PAGE). Western blotting was carried out using a horseradish peroxidase (HRP)-conjugated anti-rabbit/mouse IgG antibody as the secondary antibody, and the GE Healthcare electrochemiluminescence (ECL) System (Cat. #RPN2236) was used for detection. Immune complexes were harvested from cells that were lysed in lysis buffer, followed by centrifugation at maximum speed for 10 min to remove cell debris. Protein G Sepharose 4 Fast Flow beads (GE Healthcare) were washed in 1 × PBS and incubated with each antibody overnight. The beads were then boiled in 1 × SDS sample buffer for 5 min before resolution on SDS-PAGE gels.

### RNA constructs and RT-qPCR

Total RNA was extracted from cells for reverse transcriptase (RT)-PCR analyses, as described previously ^38^. Reactions were run on an Applied Biosystems StepOne Real-Time PCR system (Applied Biosystems, Cat. #RR014A) using SYBR Green Master Mix (Thermo Fisher, Cat. #4368702). Expression levels were normalized to that of GAPDH mRNA. The primer sets used in this experiment are listed in Table EV4.

### Immunofluorescence microscopy

Cells were seeded onto microscope slides and incubated overnight before treatment. Cells were then washed in PBS, fixed in 4% paraformaldehyde in PBS for 10 min at room temperature and permeabilized with 0.2% Triton X-100 in PBS for 10 min at room temperature. Cells were then washed twice in PBS and blocked for 1 h with 2% BSA in PBS. Cells were incubated with primary antibodies at 4 °C overnight, washed three times with 2% BSA in PBS, and incubated with secondary antibodies (goat anti-rabbit Alexa Fluor 488 or goat anti-rabbit Alexa Fluor 594) in PBS for 2 h at 4 °C. After three washes with PBS, coverslips were mounted, and images were taken using a confocal fluorescence microscope (FV1200, Olympus).

### Statistical analysis

Significant differences between two groups were identified using the unpaired Student’s *t*-test. Multiple group comparisons were analysed using Tukey–Kramer tests. Error bars represent standard deviations (SDs). Significance was set at *p < 0.05 and **p < 0.01.

## Supplementary information

Supplementary Information.
