# Supplementary material for: Stability of tuberous sclerosis complex 2 is controlled by methylation at R1457 and R1459
Source: Sci Rep. 2020 Dec 3;10:21160. doi: 10.1038/s41598-020-78274-6 (PMC7713242; doi:10.1038/s41598-020-78274-6)
Supplement: Supplementary file 1 — Supplementary Information. [file 41598_2020_78274_MOESM1_ESM.pdf]

Stability of tuberous sclerosis complex 2 is controlled by methylation at R1457 and R1459

Seishu Gen[1], Yu Matsumoto[1], Ken-Ichi Kobayashi[1], Tsukasa Suzuki[1], Jun Inoue, & Yuji Yamamoto[1]*

1 Department of Agricultural Chemistry, Faculty of Applied Bioscience, Tokyo University of Agriculture, 1-1-1 Sakuragaoka, Setagaya-ku, Tokyo, 156-8502, JAPAN

* To whom correspondence should be addressed:
Laboratory of Nutritional Biochemistry, Department of Applied Biological Chemistry, Faculty of Applied Bioscience Tokyo University of Agriculture
1-1-1 Sakuragaoka, Setagaya-ku, Tokyo 156-8502, Japan

Tel/ Fax: 03-5477-2320

Email: yujiya@nodai.ac.jp

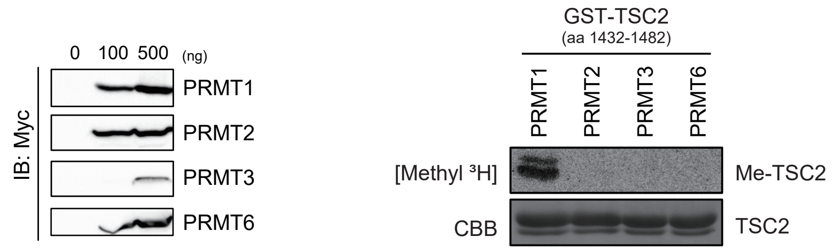

Supplementary Fig. 1.

(Left) Expression of PRMTs. Expression of PRMT used in the in vitro methylation assays as confirmed by western blotting analysis (IB). Myc-tagged PRMTs (PRMT1, PRMT2, PRMT3 and PRMT6) and Human PRMT1, PRMT2, PRMT3, and PRMT6 were amplified from HeLa cDNA using polymerase chain reaction (PCR) and inserted into pRK7-N-Myc vectors. Each vector was transfected into HEK293T cells and used for further analysis.

(Right) ***In vitro methylation assay*** **of TSC2 peptides.** Identification of PRMT responsible for TSC2 methylation by incubation of Myc-PRMTs (PRMT1, PRMT2, PRMT3, or PRMT6) with GST-TSC2 peptide (aa 1432–1482) in the presence of [3H]-SAM, followed by detection with autoradiography.

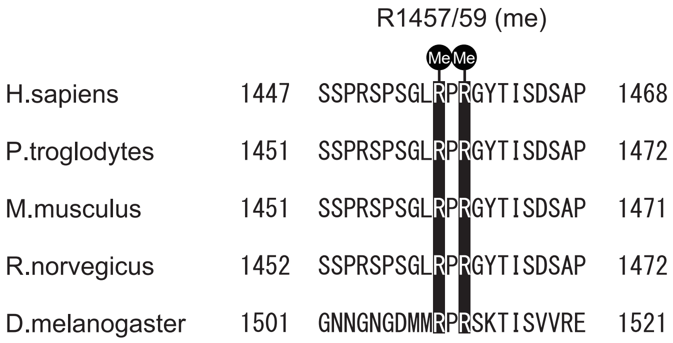

Supplementary Fig. 2. Amino acid sequence alignment of TSC2 in various species. The consensus Akt phosphorylation motif (RxRxxS/T) is highly conserved among H. sapiens, P. troglodytes, M. musculus, R. norvegicus and D. melanogaster.

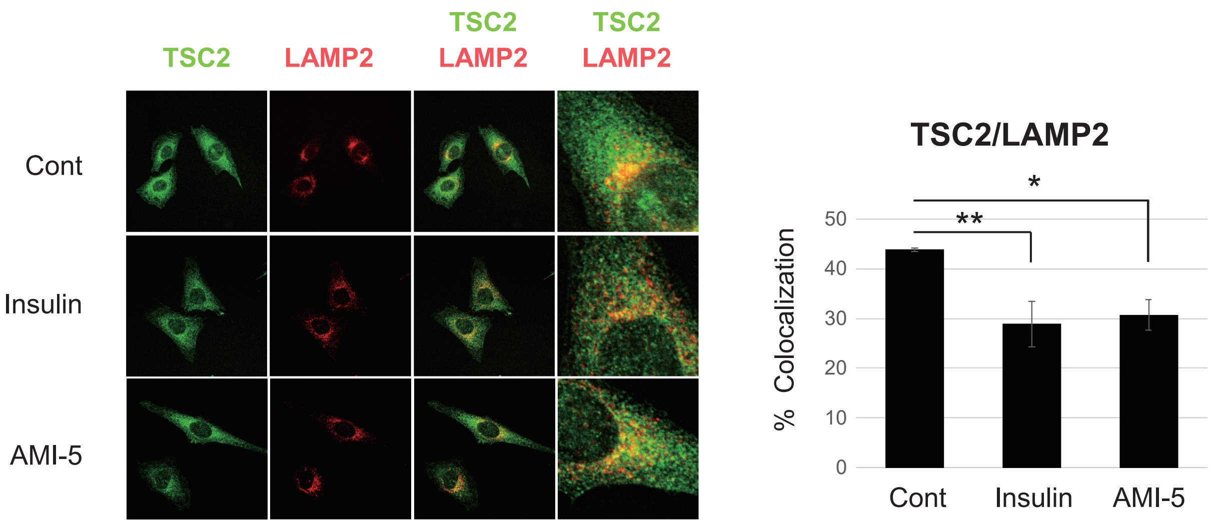

Supplementary Fig. 3. TSC2 localization by hypomethylated.

Phosphorylation levels were confirmed by analyzing intracellular localization of TSC2 on lysosomes. HeLa cells were treated with insulin or the PRMT1 inhibitor AMI-5. Endogenous TSC2 (green) and the lysosome marker LAMP2 (red) were detected using a fluorescence microscope. The right panel shows a marginal image of TSC2 and lysosomes. Percent colocalization are graphed as a mean ± SEM (right). Error bars indicate the SD from two independent experiments; $^{**}P < 0.01$, $^{*}P < 0.05$.

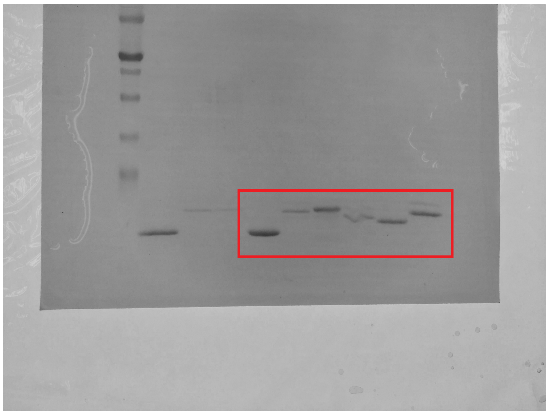

CBB

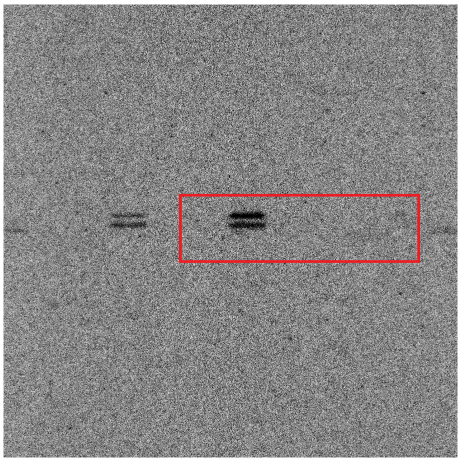

[Methyl ^{3}H]

Supplementary Fig. 4. Full-size blots to Figure 1B.

Red boxes indicate the cropped blots presented in Figure 1B.

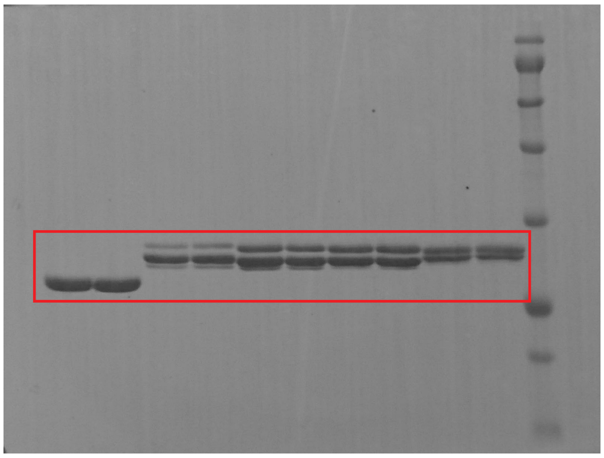

CBB

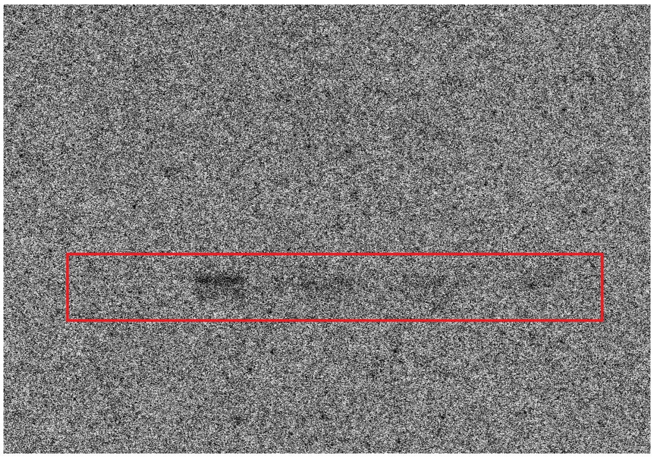

[Methyl ^{3}H]

Supplementary Fig. 5. Full-size blots to Figure 1D.

Red boxes indicate the cropped blots presented in Figure 1D.

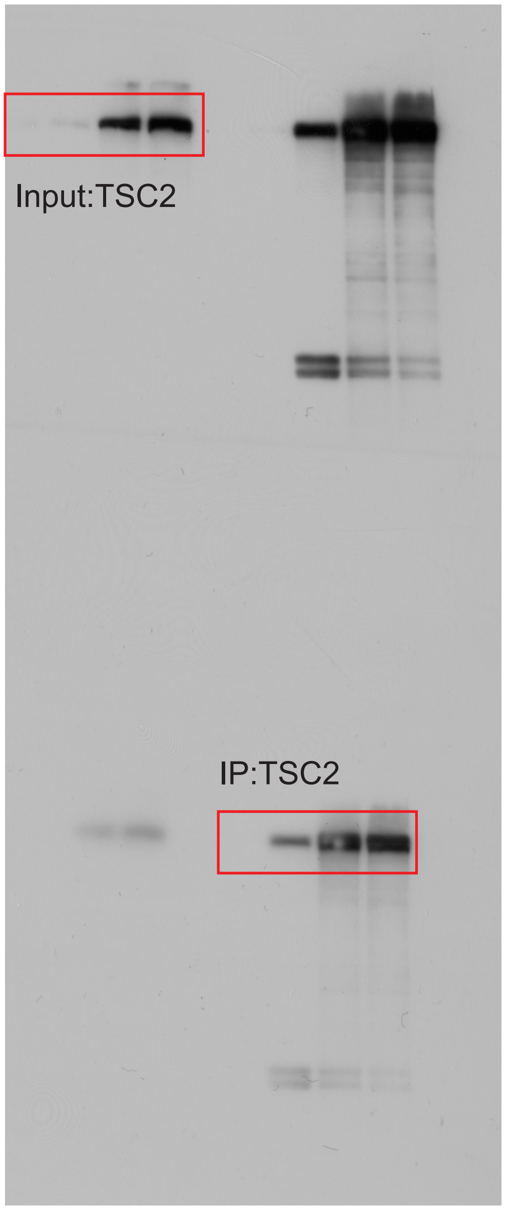

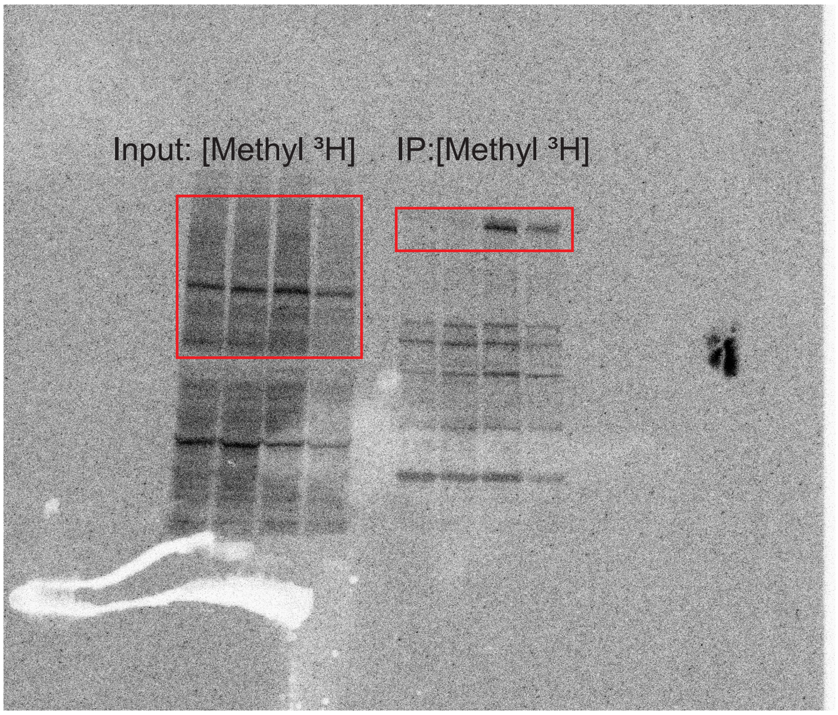

Supplementary Fig. 6. Full-size blots to Figure 2A.

Red boxes indicate the cropped blots presented in Figure 2A.

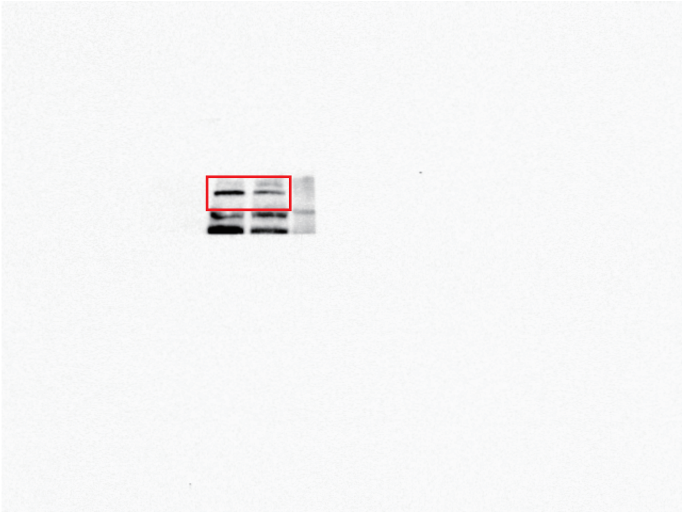

IB: ADMA

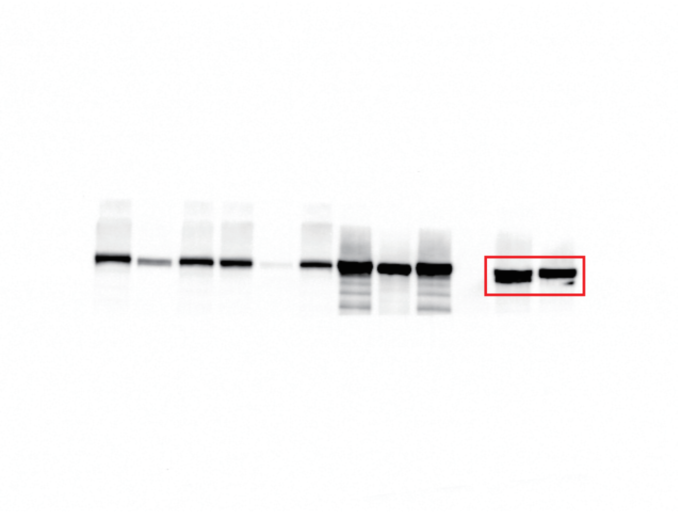

IB: TSC2

Supplementary Fig. 7. Full-size blots to Figure 2B.

Red boxes indicate the cropped blots presented in Figure 2B.

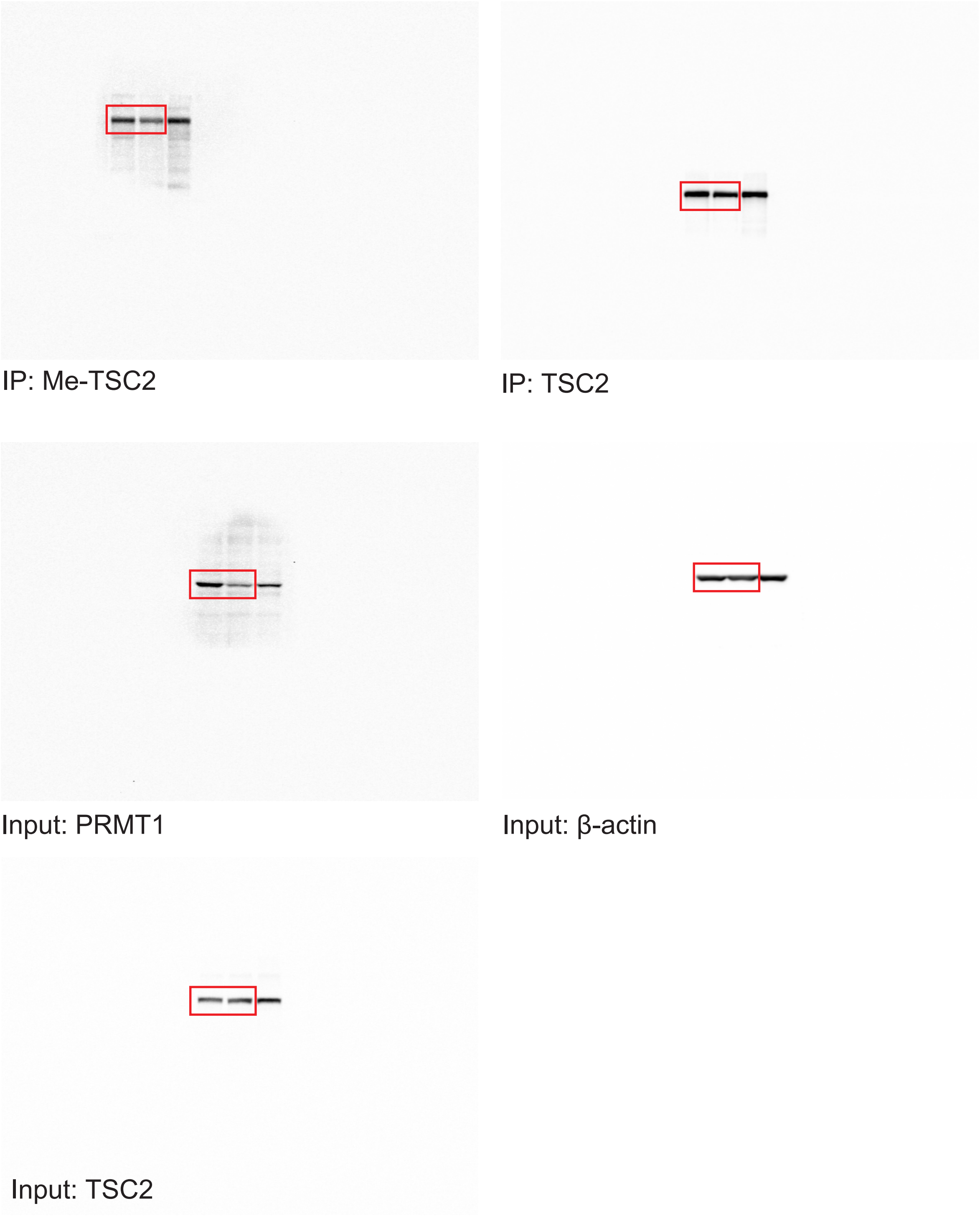

Supplementary Fig. 8. Full-size blots to Figure 2C.

Red boxes indicate the cropped blots presented in Figure 2C.

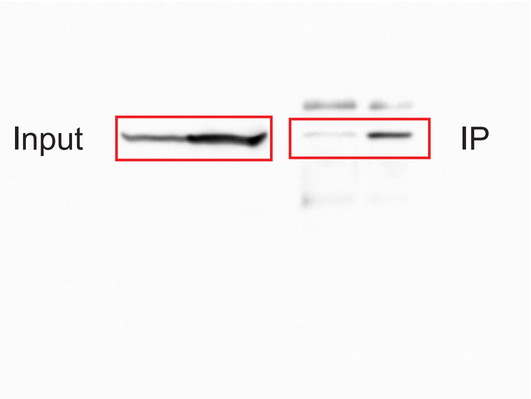

Myc-PRMT1

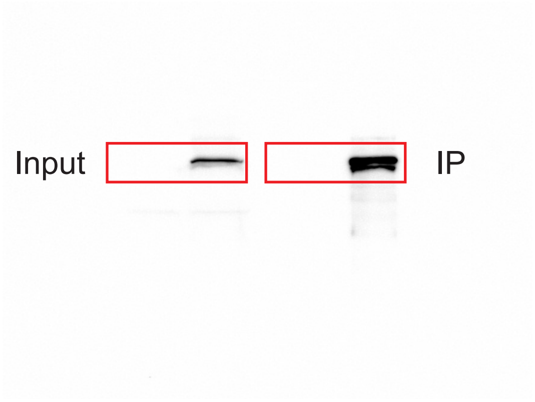

HA-TSC2

Supplementary Fig. 9. Full-size blots to Figure 2D.

Red boxes indicate the cropped blots presented in Figure 2D.

Left

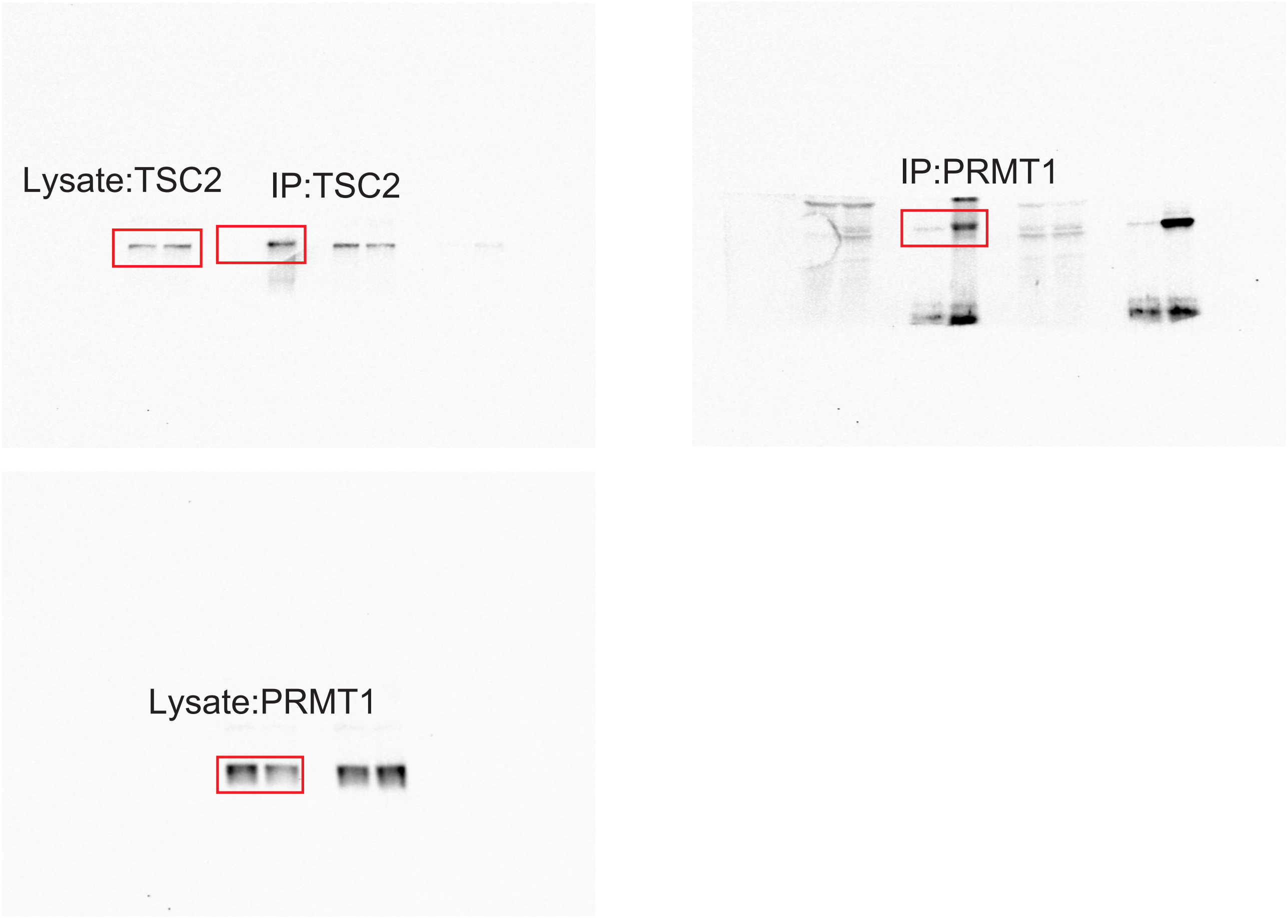

Right (IP:PRMT1)

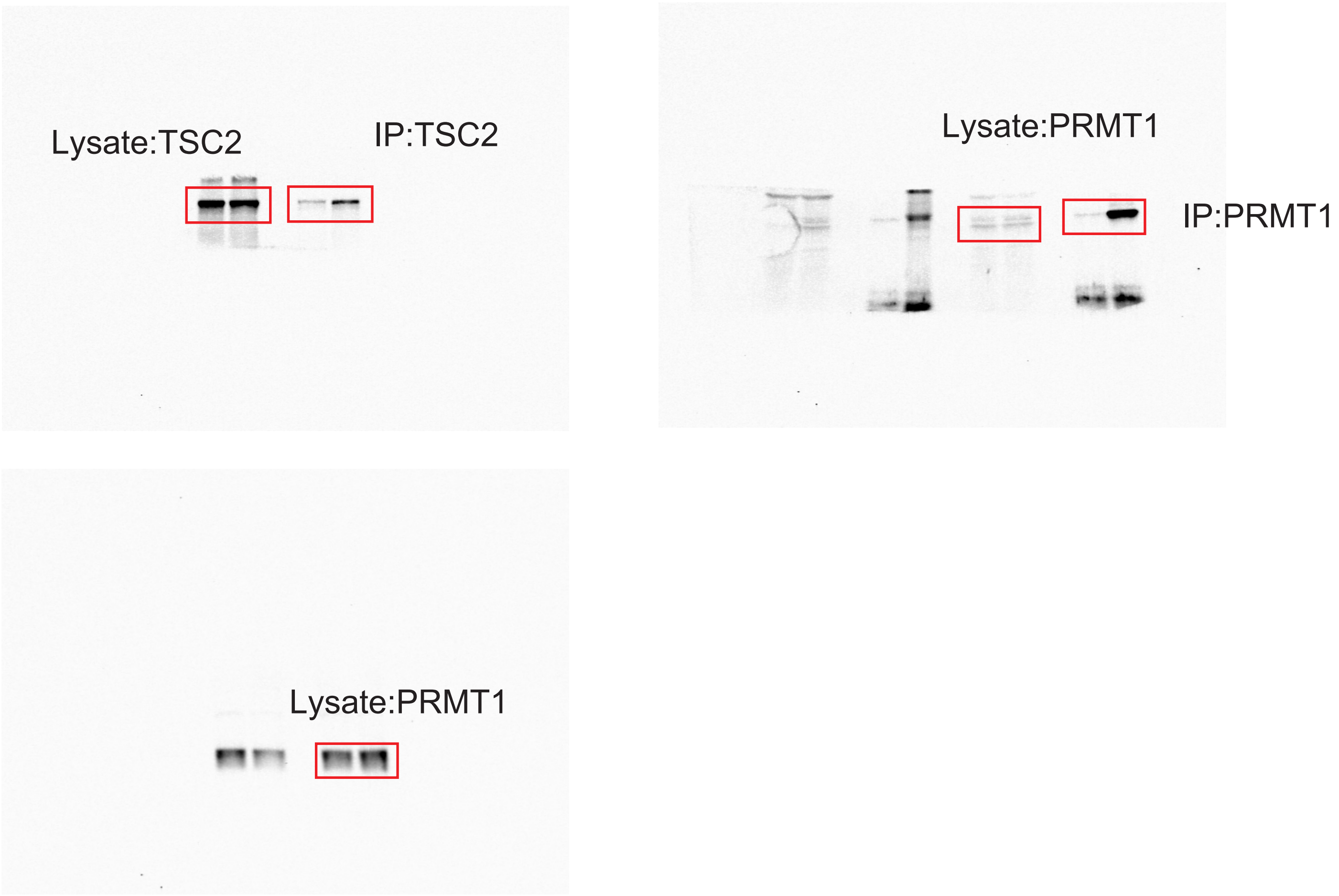

Supplementary Fig. 10. Full-size blots to Figure 2E.

Red boxes indicate the cropped blots presented in Figure 2E.

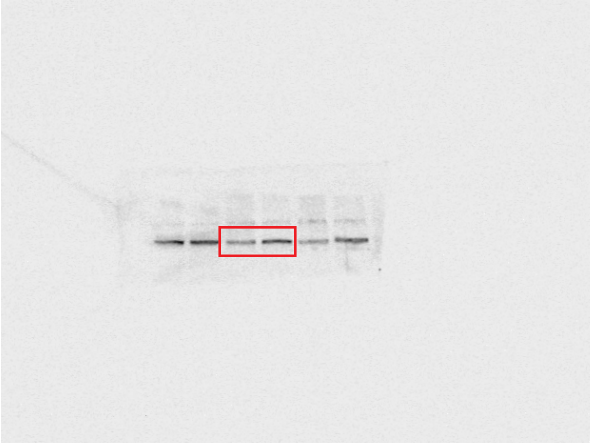

pTSC2

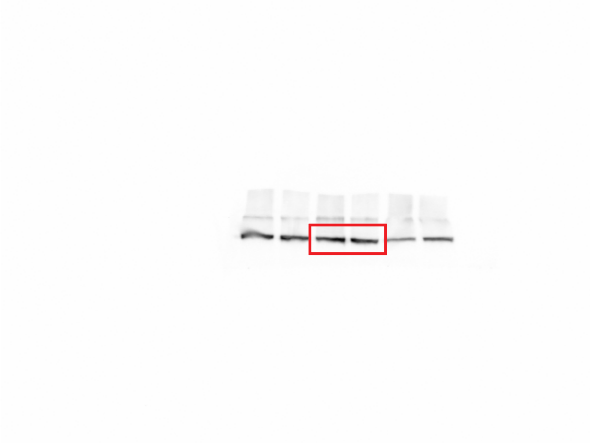

TSC2

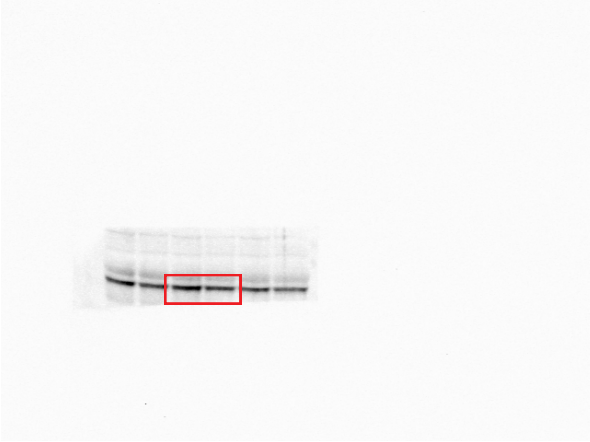

AKT

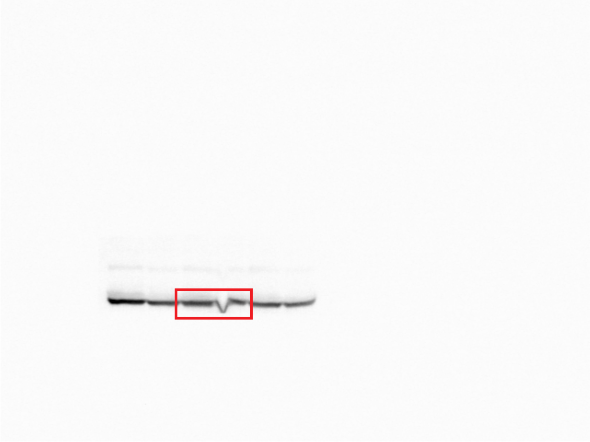

pAKT

Supplementary Fig. 11. Full-size blots to Figure 3B.

Red boxes indicate the cropped blots presented in Figure 3B.

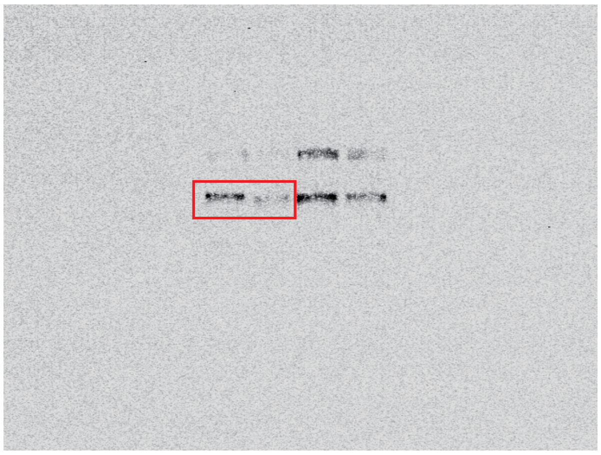

TSC2

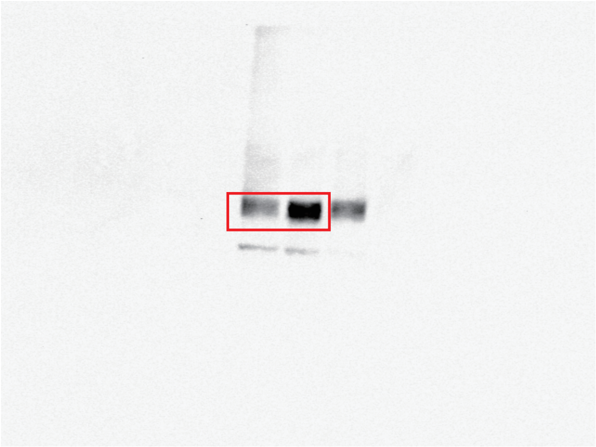

p-TSC2

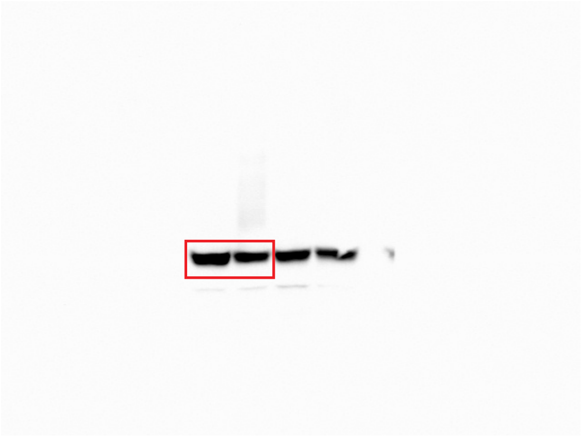

AKT

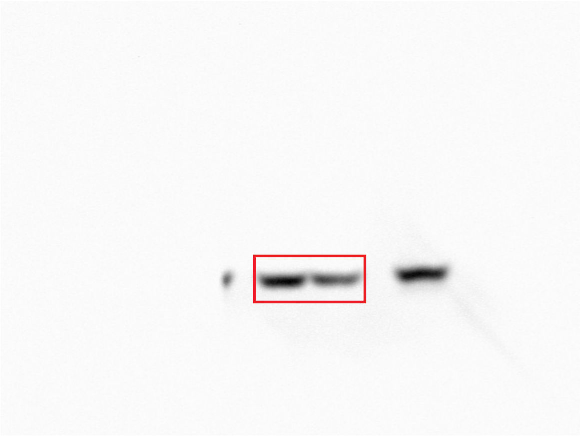

p-AKT

Supplementary Fig. 12. Full-size blots to Figure 3C.

Red boxes indicate the cropped blots presented in Figure 3C.

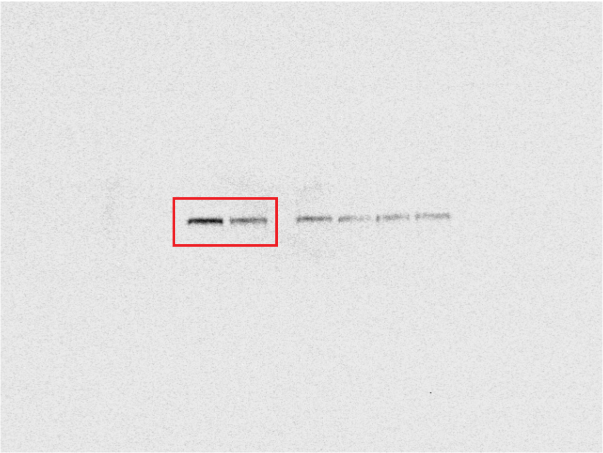

TSC2

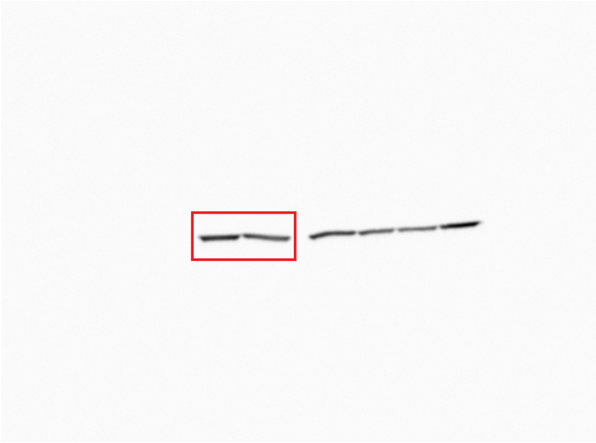

β-actin

Supplementary Fig. 13. Full-size blots to Figure 4A.

Red boxes indicate the cropped blots presented in Figure 4A.

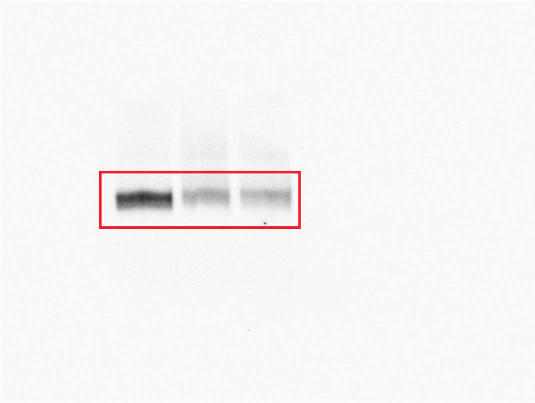

TSC2

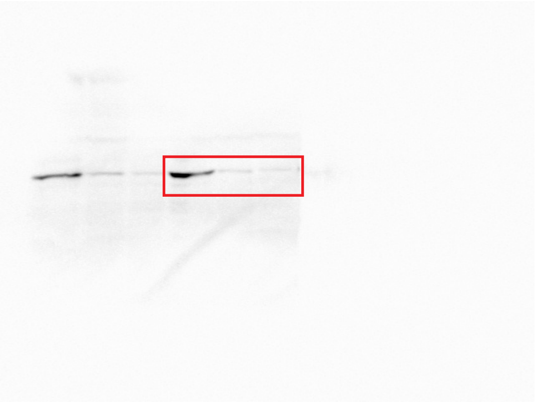

PRMT1

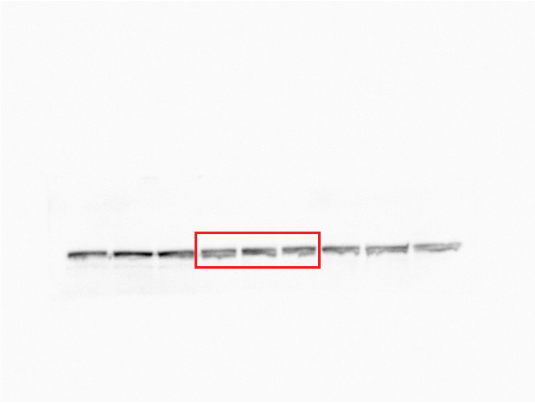

TSC1

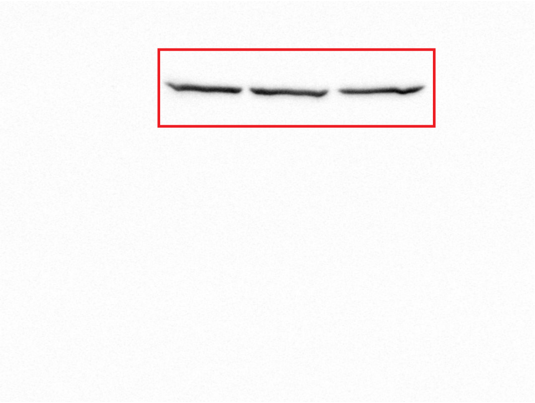

β-actin

Supplementary Fig. 14. Full-size blots to Figure 4C.

Red boxes indicate the cropped blots presented in Figure 4C.

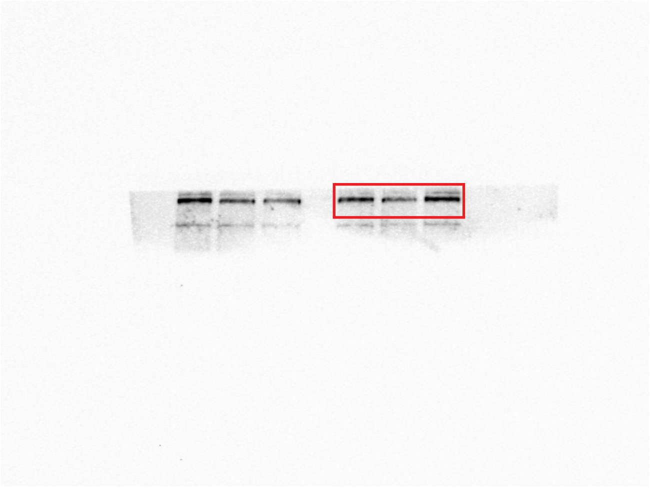

TSC2

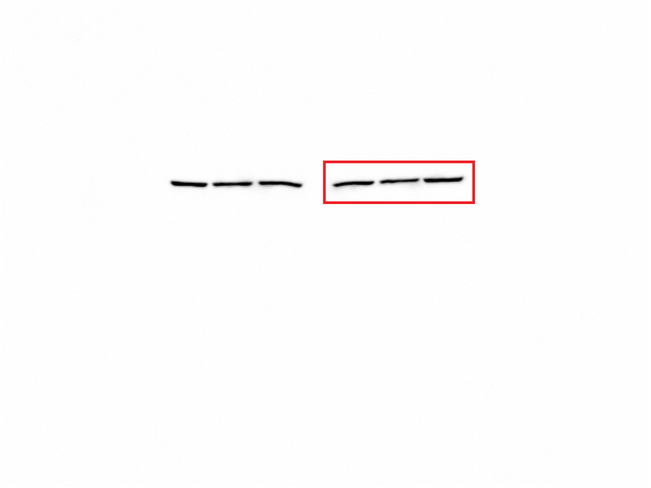

β-actin

Supplementary Fig. 15. Full-size blots to Figure 4D.

Red boxes indicate the cropped blots presented in Figure 4D.

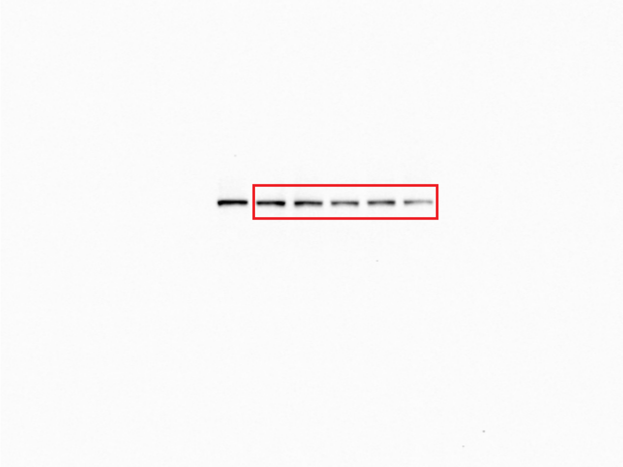

TSC2

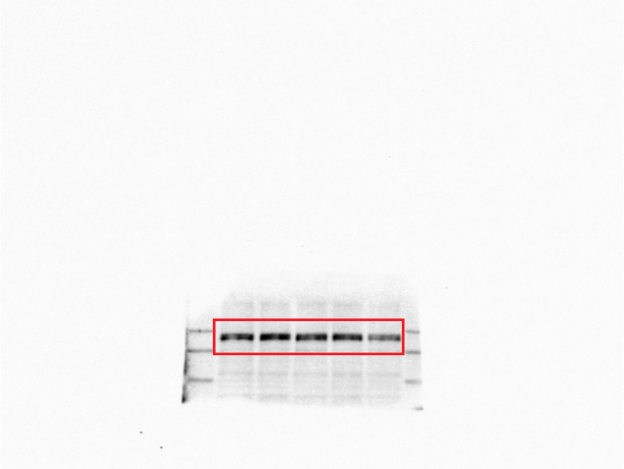

TSC1

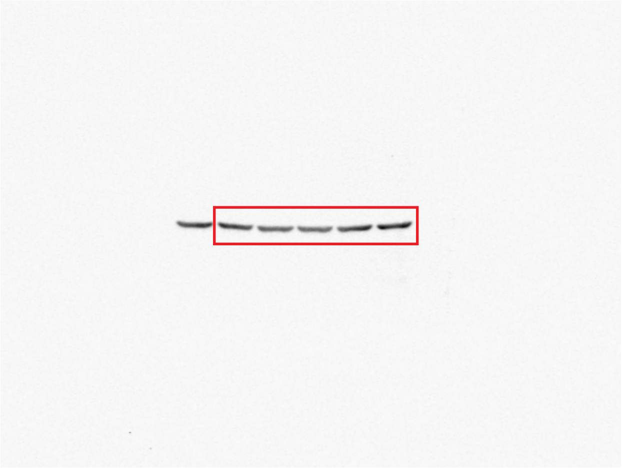

β-actin

Supplementary Fig. 16. Full-size blots to Figure 4E.

Red boxes indicate the cropped blots presented in Figure 4E.

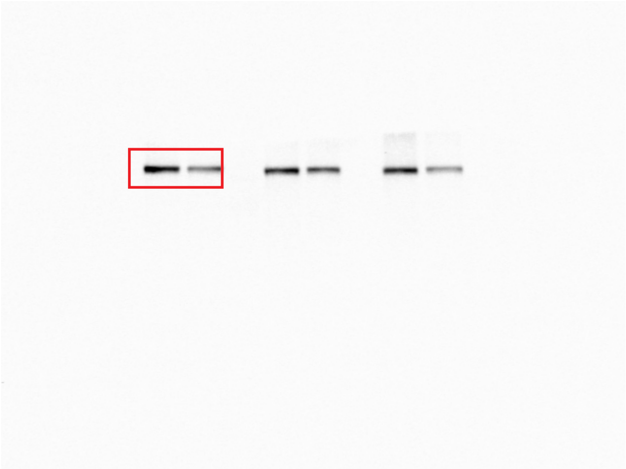

TSC2

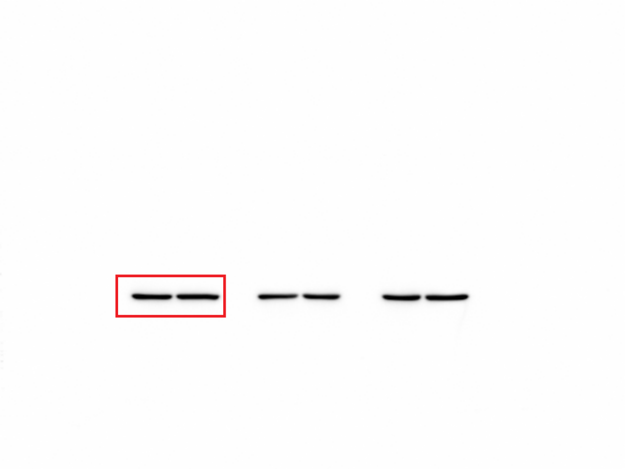

β-actin

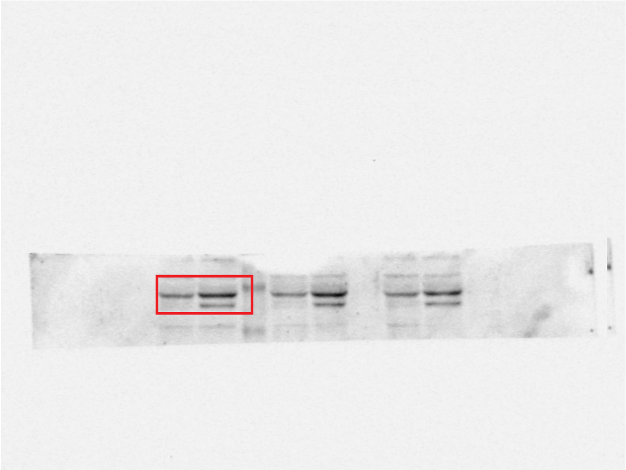

pS6K (Thr389)

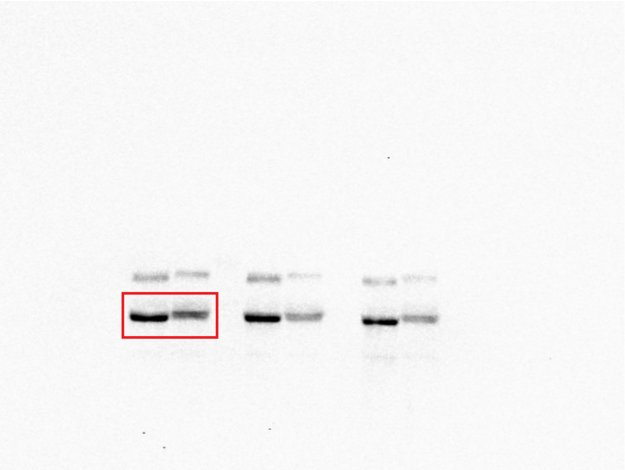

S6K

Supplementary Fig. 17. Full-size blots to Supplementary Fig. 4F.

Red boxes indicate the cropped blots presented in Supplementary Fig. 4F.

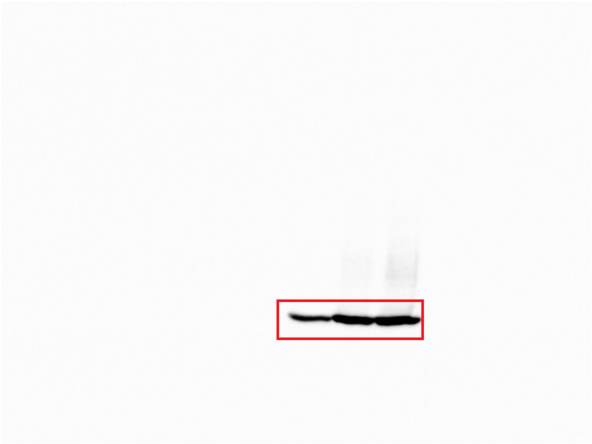

Myc-PRMT1

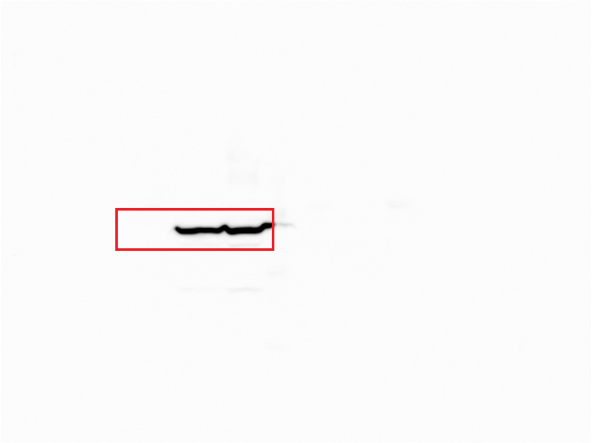

Myc-PRMT2

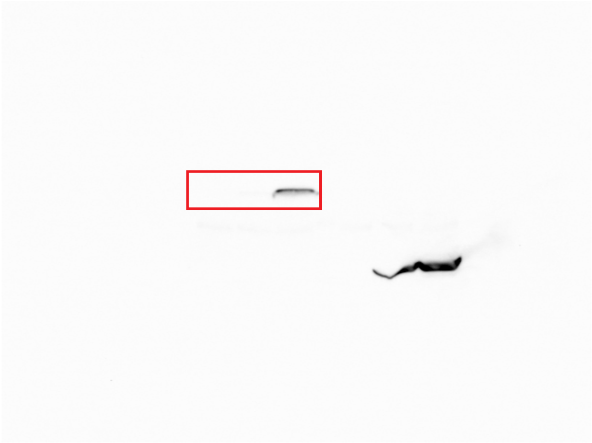

Myc-PRMT3

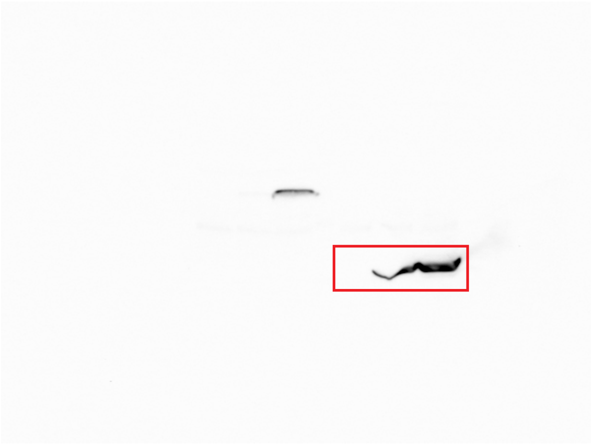

Myc-PRMT6

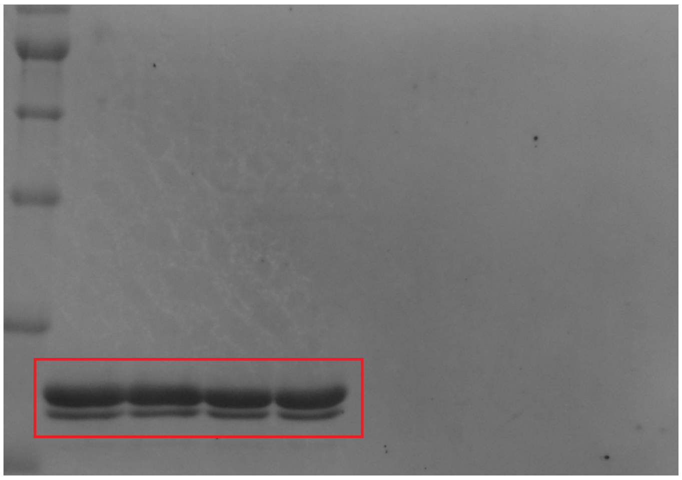

CBB

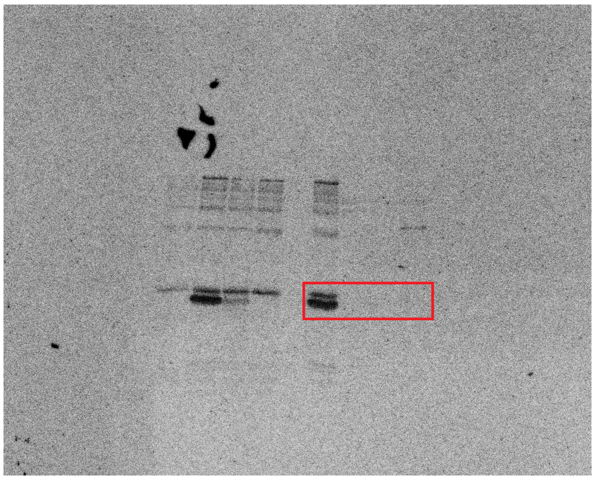

[Methyl ^{3}H]

Supplementary Fig. 18. Full-size blots to Supplementary Fig. 1.

Red boxes indicate the cropped blots presented in Supplementary Fig. 1.

Table EV1.

Specific primers used for Myc-PRMT plasmids.

1. human Myc-PRMT1	Primer (forward) 5’	- ATGGATCCATGGCGGCAGCCGA -3’
	Primer (reverse) 5’	- ATATCGATTCAGCGCATCCGGTAGTC -3’
2. human Myc-PRMT2	Primer (forward) 5’	- ATGGATCCATGGCAACATCAGGTGACTG -3’
	Primer (reverse) 5’	- ATATCGATTCATCTCCAGATGGGGAAGA -3’
3. human Myc-PRMT3	Primer (forward) 5’	- ATGGATCCATGTGTCTGCTCGTTAGCGTCAGG -3’
	Primer (reverse) 5’	- ATATGATTCACTGGAGACCATAAGTTGAGTT -3’
4. human Myc-PRMT6	Primer (forward) 5’	- ATGGATCCATGTCYCGCAGCCCAAGAAA -3’
	Primer (reverse) 5’	- ATATCGATTCAGTCCTCCATGGCAAAG -3’

Table EV2.

Specific primers used for shRNA constructs.

PRMT1 shRNA #1 :	5’ -CCGGGGACATGACATCCAAAGATTACTCGAGTAATCTTTGGATGTCATGTCCTTTTTC -3’
PRMT1 shRNA #2 :	5’ -CCGGGCAACTCCATGTTTCATAACCCTCGAGGGTTATGAAACATGGAGTTGCTTTTTC -3’

Table EV3.

Specific primers used for recombinant GST-TSC2 protein.

1. GST-TSC2 1432-1482 aa		
	Primer (forward) 5’	- ATGAATTCTAGGCGAAGACAGTCGGG -3’
	Primer (reverse) 5’	- ATAAGCTTTCATCTGCTCTTTAAGGCGTCC -3’
2. GST-TSC2 for Point Mutation at R1457A		
	Primer (forward) 5’	- GGCCTCGCACCCCGAGGTTACACCATC -3’
	Primer (reverse) 5’	- TCGGGGTGCGAGGCCACTGGGCGAGCG -3’
3. GST-TSC2 for Point Mutation at R1459A		
	Primer (forward) 5’	- CGGCCCGCAGGGTTACACCATCTCCGAC -3’
	Primer (reverse) 5’	- GTAACCTGCGGGCCGGAGGCCACTGGG -3’
4. GST-TSC2 for Point Mutation at R1457A/R1459A		
	Primer (forward) 5’	- CTCGCACCCGCAGGTTACACCATG -3’
	Primer (reverse) 5’	- ACCTGCGGGTGCGAGGCCACTGGG -3’
5. GST-TSC2 for Point Mutation for R1470STOP		
	Primer (forward) 5’	- CCATCATGAAGGGGCAAGAGAGTAGAG -3’
	Primer (reverse) 5’	- GCCCCTTCATGATGGGGCCGAGTCGGA -3’
6. GST-TSC2 636- 685 aa	Primer (forward) 5’	- ATGGATCCGCCAAGGACGGCTGG -3’
	Primer (reverse) 5’	- AAGCTTGGGCACGGACCCCAG -3’
7. GST-TSC2 726- 775 aa	Primer (forward) 5’	- ATGGATCCGCCAAGGACGGCTGG -3’
	Primer (reverse) 5’	- AAGCTTAGAGATTAATGCTGTCAGCACTG -3’
8. GST-TSC2 1094- 1143 aa	Primer (forward) 5’	- TCGAGCTCCAGCCCC -3’
	Primer (reverse) 5’	- GTCCAGGGCGCCAAC -3’
9. GST-TSC2 1330- 1378 aa	Primer (forward) 5’	- ATGGATCCGATGCCTACAGCAGGTCGTC -3’
	Primer (reverse) 5’	- AAGCTTGGGCTGGAAGGAGAGGTC -3’

Table EV4.

The following specific primers were used for qRT–PCR analysis.

human TSC2 primer (forward) :	5’ - AACCCCAGTTTCGTGTTCC-3’
human TSC2 primer (reverse) :	5’ -GGGATTGGCTTGTTTGACTCG-3’
